# Bioinformatic prediction of G protein-coupled receptor encoding sequences from the transcriptome of the foreleg, including the Haller’s organ, of the cattle tick, *Rhipicephalus australis*

**DOI:** 10.1371/journal.pone.0172326

**Published:** 2017-02-23

**Authors:** Sergio Munoz, Felix D. Guerrero, Anastasia Kellogg, Andrew M. Heekin, Ming-Ying Leung

**Affiliations:** 1 The University of Texas at El Paso, Bioinformatics Program, El Paso, Texas, United States of America; 2 USDA-ARS, Knipling Bushland US Livestock Insect Research Laboratory, Kerrville, Texas, United States of America; University of Michigan, UNITED STATES

## Abstract

The cattle tick of Australia, *Rhipicephalus australis*, is a vector for microbial parasites that cause serious bovine diseases. The Haller’s organ, located in the tick’s forelegs, is crucial for host detection and mating. To facilitate the development of new technologies for better control of this agricultural pest, we aimed to sequence and annotate the transcriptome of the *R*. *australis* forelegs and associated tissues, including the Haller's organ. As G protein-coupled receptors (GPCRs) are an important family of eukaryotic proteins studied as pharmaceutical targets in humans, we prioritized the identification and classification of the GPCRs expressed in the foreleg tissues. The two forelegs from adult *R*. *australis* were excised, RNA extracted, and pyrosequenced with 454 technology. Reads were assembled into unigenes and annotated by sequence similarity. Python scripts were written to find open reading frames (ORFs) from each unigene. These ORFs were analyzed by different GPCR prediction approaches based on sequence alignments, support vector machines, hidden Markov models, and principal component analysis. GPCRs consistently predicted by multiple methods were further studied by phylogenetic analysis and 3D homology modeling. From 4,782 assembled unigenes, 40,907 possible ORFs were predicted. Using Blastp, Pfam, GPCRpred, TMHMM, and PCA-GPCR, a basic set of 46 GPCR candidates were compiled and a phylogenetic tree was constructed. With further screening of tertiary structures predicted by RaptorX, 6 likely GPCRs emerged and the strongest candidate was classified by PCA-GPCR to be a GABA_B_ receptor.

## Introduction

The cattle tick, *Rhipicephalus (Boophilus) australis*, vectors the microbial agents that cause anaplasmosis and babesiosis in cattle [1]. Infected cattle usually experience a decrease in weight, reduced milk production, and even death, especially in animals never exposed to cattle ticks and, thus, immunologically naïve. Therefore, tick control strategies are an essential part of livestock management practices and the application of acaricides remains central to tick control [2]. Unfortunately, resistance to new acaricides has historically appeared in ticks within a relatively few years after acaricide introduction [3]. Novel acaricides or novel control technologies are needed to maintain successful tick control programs. The identification of olfactory or neural receptors that regulate tick metabolism or behavior might facilitate the development of new acaricides or repellents, as these receptors could be targeted by novel compounds. While receptor identification can be assisted by genomic and transcriptomic data, tick genomic resources are lacking, with only the genome of the Prostriate deer tick, *Ixodes scapularis*, available for gene data mining. Transcriptome studies are more accessible, and recent studies have provided new insights regarding tick neurosensorial biology, particularly studies of the synganglion transcriptome. Bissinger et al. [4] sequenced the transcriptome of synganglia from the American dog tick, *Dermacentor variabilis*, identifying and quantifying the expression of several neuropeptides, receptors, and transporters in that neural tissue. Christie et al. [5] mined transcriptome datasets in GenBank to identify novel allatostatins and allatostatin precursor neuropeptides in the tick, *Amblyomma variegatum*. Guerrero et al. [6] utilized bioinformatic approaches to predict G protein-coupled receptor (GPCR) encoding sequences in the synganglion transcriptome of *Rhipicephalus microplus*.

GPCRs constitute a large protein family of receptors that sense molecules and activate signal transduction pathways, ultimately regulating various cellular responses. GPCRs are found only in eukaryotes [7] and are common drug targets in humans [8]. For example, the large group of class A (rhodopsin-like) receptors are recognized as targets for the development of novel drugs [9]. Ligands that bind to GPCRs include odorants, hormones, pheromones, and neurotransmitters. The ligand-GPCR interaction activates G-proteins and triggers specific intracellular events. Based on their functional similarity or homology, there are several classification systems used to divide the GPCR superfamily [10–11]. The GPCR database GPCRDB [12] available at http://www.gpcr.org/7tm/, has 53 curated GPCRs from the genome of the deer tick, *I*. *scapularis*, and at least this number likely exists in *R*. *australis*. Investigations in understudied organisms such as ticks produce high numbers of orphan genes, which lack detectable sequence homology to genes in pre-existing databases [13]. This complicates studies seeking to comprehensively identify transcripts and proteins in these organisms. Nevertheless, several GPCRs have been identified and studied in *R*. *microplus* including a serotonin receptor [14], a Type-1 tyramine receptor [15], and a leukokinin-like receptor [16]. In congruence with research priorities aimed at developing novel cattle tick control technologies, we initiated a study of genes expressed in the forelegs of *R*. *australis* with a specific priority of identifying and predicting the GPCR transcripts expressed in tissues and organs associated with the foreleg.

The Haller’s organ is a sensory structure on the dorsal surface of the tarsi of the first pair of legs. The Haller’s organ consists of a posterior capsule containing numerous sensilla exposed to the exterior via a small aperture and an open anterior pit located distal to the posterior capsule that contains both olfactory and gustatory sensilla [17]. Sensilla in this organ possess a wide array of sensory capabilities, responding to various olfactory, mechanical, and environmental stimuli [18]. Sensilla from the Haller’s organ of the Cayenne tick, *Amblyomma cajennense*, were found to respond to compounds related to sexual attraction and questing behavior [19–20]. However, the roles of the Haller’s organ in tick behavior are not clear and species-dependent responses might exist [21]. An analysis of a comprehensive transcriptome dataset for the Haller's organ has not been reported. Thus, we sequenced and annotated the transcriptome of adult *R*. *australis* forelegs, which include the Haller's organ, using a Titanium 454 pyrosequencing approach, optimized for long read length. However, as with many short read next-generation sequence-based datasets, many partial transcript sequences remained in the assembled database. Thus, an approach that only examined full length transcripts for GPCR-like transcripts would likely produce an incomplete result. In addition, sequence diversity between related GPCRs can be extensive, making acquisition of a comprehensive GPCR database difficult if only sequence-based approaches are utilized. However, GPCRs possess a conserved structural motif consisting of seven transmembrane helices. This trait can be exploited to improve bioinformatic predictions of GPCRs from non-model organisms. For example, Wistrand et al. [22] used structural-based proteome searching and detected 102 protein-coding sequences in *Caenorhabditis elegans* that were identified as potentially novel GPCRs that showed no significant sequence similarity to known GPCRs. Also, Zamanian et al. [23] identified 66 Platyhelminth-specific *Rhodopsin*-like orphan family 1 proteins with classical *Rhodopsin* motifs but no significant sequence similarity to known GPCRs. They also identified a large number of *Rhodopsin* receptors that lacked meaningful sequence similarity to other GPCRs. Guerrero et al. [6] discovered 27 putative GPCRs from the synganglion of the tick, *R*. *microplus*, using structural-based approaches that were not detected by sequence similarity-based approaches. Thus, for our study, both structurally-based and sequence-based approaches were used to predict GPCRs from the *R*. *australis* foreleg transcriptome and classify them into GPCR families based on the human GPCR classification model [24].

## Methods

**Fig 1:**
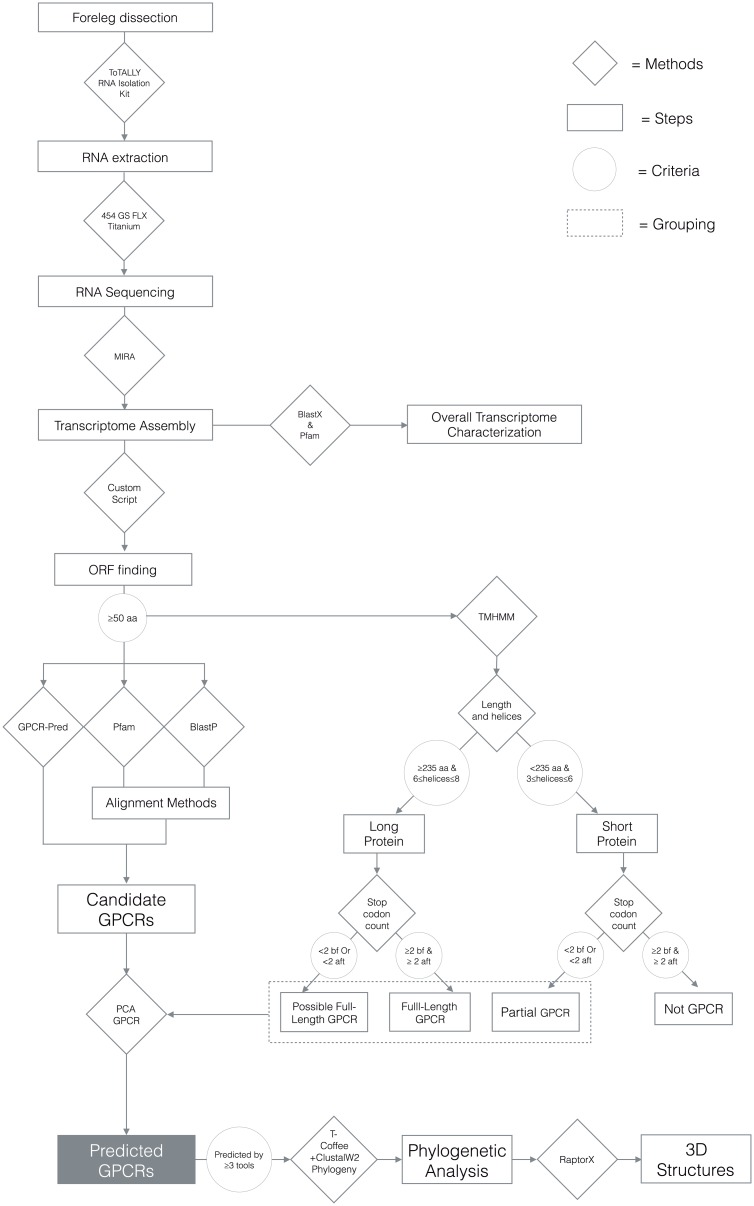
Overall workflow of the study. Visual representation of all steps of the study, from wet-lab procedures to bioinformatic analysis. Steps taken and results are represented by rectangles, methods by rhombuses and criteria by circles.

This study was conducted in two stages. The first stage involved wet-lab procedures of RNA extraction, sequencing, and assembly. This was followed by computational analyses to characterize the overall transcriptome and subsequently predict likely GPCR candidates using a combination of sequence similarity searches, alignment-free methods, and structurally-based approaches. Fig 1 displays the overall workflow.

### Tick materials

*R*. *australis* ticks were obtained from the Australian NRFS laboratory strain reared upon Hereford cattle at the Biosecurity Tick Colony, Animal Research Institute, Yeerongpilly, Queensland, Australia [25]. Pairs of the entire first legs including the tarsal segment containing the Haller's organ were dissected from 162 female and 10 male unfed adult ticks immobilized under phosphate-buffered saline (pH 7.0), excising between the trochanter and femur. Upon dissection, the materials were immediately placed in a pre-chilled 1.5 ml microcentrifuge tube submerged in dry ice. When all the ticks were dissected, RNA*later* ICE (Life Technologies, Grand Island, NY, USA) was added according to the supplier's protocol and the materials were shipped on dry ice to the United States and stored at -80°C until processed.

### RNA extraction, sequencing, and assembly

Total RNA was extracted using the ToTALLY RNA Isolation Kit (Life Technologies) per manufacturer's recommendation after thawing on ice, centrifugation and removal of excess RNA*later* ICE. The optional lithium chloride precipitation step suggested by the kit protocol was used to help remove genomic DNA from the RNA. Following agarose gel electrophoretic analysis of the RNA, RNA integrity was good but genomic DNA was detected in the samples, thus, the TURBO DNA-*free* kit (Life Technologies) was used per manufacturer's recommendation to enzymatically remove the genomic DNA. The MicroPoly(A)Purist Kit (Life Technologies) was used to purify polyadenylated RNA and the Just cDNA Kit (Stratagene, La Jolla, CA USA) used to prepare cDNA for sequencing.

Sequencing was done using a 454 GS FLX Titanium platform by massively parallel pyrosequencing as described by the manufacturer [26]. Sequence assembly was performed using the MIRA assembler with the EST option [27]. The raw 454 reads were submitted to the National Center for Biotechnology Information's Short Read Archive under Accession Number SRA052633. All resulting contigs and unassembled singletons were collectively called unigenes. Each unigene received a unique number preceded by the prefix “lcl”. For the bioinformatic analysis each unigene was then mapped to a shorter name consisting of the prefix “athaller” followed by +1 incremental numbering (S1 File). This dataset was then submitted to the Transcriptome Shotgun Assembly Database (TSA) at GenBank (Accession Number GEMR00000000).

### Transcriptome characterization using Blastx and Pfam

Unigenes were annotated via sequence similarity searches using Blastx with default parameters against the UniRef100 database [28–29], where hits with E-value < 1e-06 were considered significant. Each unigene was analyzed with a custom open reading frame (ORF)-finding script (S2 File) which was built by utilizing the .translate and .reverse codes in Biopython library (http://biopython.org/DIST/docs/tutorial/Tutorial.html#sec360). All 6 possible reading frames were examined, outputting any resulting protein sequences ≥ 50 amino acids in length. ORFs with less than 50 amino acids were deemed extremely unlikely to encode receptors or GPCRs and were discarded. As some unigenes had several possible ORFs, sequence length, strand and frame were added to the unigene IDs to make the ORF IDs unique. We queried the ORF sequences against the web version of the Pfam database (http://pfam.xfam.org/) for common domains. From the Pfam output, we created a clan/superfamily distribution to assess the proportion of each of them in the context of the whole transcriptome.

### Bioinformatic identification of GPCR candidates

To predict which unigenes are likely to encode a GPCR. We applied a combination of different bioinformatic tools, including both sequence alignment and alignment-free methods, as well as structurally based approaches.

The alignment methods comprise Blastx, Blastp [30], and Pfam [31]. Using the Blastx results already obtained in the transcriptome characterization above, we searched for the word “receptor” in all significant hits. The resulting list was manually screened by searching for the terms compiled in Table 1 fetched from the QuickGo-EMBL-EBI portal using the ID “GO:0004930” (available at https://www.ebi.ac.uk/QuickGO/) to identify those known to have GPCR activity. For the Blastp analysis we downloaded an executable version from the NCBI/BLAST website repository (ftp://ftp.ncbi.nlm.nih.gov/blast/executables/blast+/LATEST/) to a Dell Optiplex 790 computer with 8Gb of memory running the Linux Centos 7 operating system. We used Blastp with default parameters (BLOSUM62 matrix, Gap costs 11:1) to search the translated ORF sequences against the annotated GPCRs from the *I*. *scapularis* genome assembly [32] and the *R*. *microplus* and *R*. *australis* [6] synganglion transcriptomes, as well as *in silico* predicted neuropeptide and neurohormone GPCR datasets from the spider mite *Tetranychus urticae* [33], and a group of other chelicerates including *Stegodyphus mimosarum*, *Latrodectus hesperus*, *Parasteatoda tepidariorum*, *Acanthoscurria geniculata*, *Mesobuthus martensii*, and *Dermatophagoides farinae* [34]. As with the Blastx results, hits were searched for the terms in Table 1 in all significant hits with E-value < 1e-06 and manually screened by searching the UniprotKB database (www.uniprot.org). The entire ORF sequence dataset was also screened using the web version of the Pfam database (http://pfam.xfam.org/) for common domains, seeking ORFs having a hit to any of the 7tm families and examining the clan description for those indicated to be GPCRs.

**Table 1:** GPCR Synonyms (GO:0004930) and terms associated with GPCR activity.

G-protein coupled receptor activity, unknown ligand
Mas proto-oncogene receptor activity
Orphan G protein coupled receptor activity
Orphan GPCR activity
RDC1 receptor activity
Super conserved receptor expressed in brain receptor activity
Epstein-Barr Virus-induced receptor activity
SREB receptor
EBV-induced receptor
Orphan G-protein coupled receptor activity
Receptor activity, G-protein coupled
G protein coupled receptor activity
G protein linked receptor activity
GPCR activity
Ligand-dependent GPCR activity

The first alignment free method used for our analysis was GPCRpred [35] a support vector machine (SVM)-based approach for predicting families and subfamilies of GPCRs using the dipeptide composition of proteins (http://osddlinux.osdd.net/prepo.php). GPCRpred provides a binary output “Y” or “N” to indicate whether each input sequence was predicted to be a GPCR and categorizes the sequence as class A (rhodopsin-like), B (secretin and adhesion), C (metabotropic glutamate), D (fungal pheromone receptors), E (cAMP receptors), or F (frizzled) according to the International Union of Basic and Clinical Pharmacology (IUPHAR) nomenclature (see http://www.guidetopharmacology.org/nomenclature.jsp). GPCRpred also indicates subfamily, if known.

TMHMM [36] is a membrane protein topology prediction method based on hidden Markov models, which is capable of predicting topology that includes transmembrane helices as well as extracellular and intracellular loops. Taking advantage of the GPCR structural characteristic, we followed an approach similar to that used by Guerrero et al [6] to identify candidate GPCRs. We used the web version at http://www.cbs.dtu.dk/services/TMHMM/ with default parameters to input our ORF dataset, and obtain output containing information on the number of predicted transmembrane helices. Since all GPCR’s have 7 transmembrane helices, one would, in principle, only need to look for ORFs with exactly seven transmembrane helices in the TMHMM output. TMHMM is also reported to be very accurate in prediction of helical regions [36]. In a set of 160 protein sequences with known topologies, TMHMM correctly predicted 97.5% of the helical regions. However, considering that some of the input sequences may be partial ORFs and that errors might have occurred during sequencing, assembly, ORF identification, or TMHMM prediction, we relaxed the criteria for selecting candidate GPCRs from the TMHMM output. Our prediction protocol (Fig 1) was based on the following principles:

1. Length of the ORF sequence. We categorized ORF sequences with lengths ≥ 235 amino acids as “long” and those < 235 amino acids “short”. The “long” sequences were assumed sufficient in length to contain the required seven transmembrane helices and the 6 loops of a GPCR, while the “short” sequences were considered unlikely to do so. The choice of 235 amino as the cut-off length was considered a conservative criterion and based on the average lengths of protein helices and loops reported by Meruelo et al. [37].
2. Predicted number of helices. All “long” ORF’s with 6–8 helices were considered GPCR candidates and retained for the next step of screening. The remaining “long” ORF’s were discarded. While ORF’s with < 235 amino acids were unlikely to encode full-length GPCRs, partial transcripts were found in our tick foreleg transcriptome. Wishing to include partial ORFs in our analysis, we retained “short” ORF’s containing 3–6 helices for further screening, and discarded the rest.
3. Number of stop codons upstream and downstream. We used the number of stop codons around an ORF as predictive of encoding a full length protein. Because sequencing and assembly errors occur in high throughput sequencing datasets, we required an ORF to have at least two stop codons both upstream and downstream in order to be classified as “full-length.” The “long” proteins with 6–8 helices, already deemed GPCR candidates in step (2), were further classified to be “full-length GPCR” if the stop codon requirement is met, and “possible full-length GPCR” if not. For the “short” ORFs with 3–6 helices retained after step (2), sequences that passed the stop codon requirement were discarded because they were likely to encode full-length proteins but without the 7 transmembrane regions required to be classified as a GPCR. However, those with less than 2 stop codons on either side might still be part of a GPCR and were therefore classified as candidate GPCRs in the category of “partial GPCRs.”

All GPCR candidates predicted by the methods described above, were analyzed by another alignment free method, PCA-GPCR, whose algorithm is based on statistical principal component analysis [38]. PCA-GPCR performs the prediction at five levels: GPCR, families, subfamilies, sub-subfamilies, and subtypes as each subtype demonstrates its own characteristic ligand binding property, coupling partners of trimeric G-proteins, and interaction partners of oligomerization. This method uses five descriptors: amino acid composition and dipeptide composition, autocorrelation descriptors, global descriptors, sequence-order descriptors, and Chou's pseudo amino acid composition descriptors. The publicly available version of PCA-GPCR (http://www1.spms.ntu.edu.sg/~chenxin/PCA_GPCR/) limits the number of sequences per submission, making it impractical to analyze our entire ORF dataset with over 40,000 sequences. Thus, we ran PCA-GPCR only on the candidate GPCRs predicted by at least one of the other tools described above.

The positive results from all bioinformatic tools were then compiled and compared against one another. Using the online Venny 2.1 program at bioinfogp.cnb.csic.es/tools/venny/, a Venn diagram was constructed to reflect the number of predicted GPCRs made by the different combinations of tools and how these predictions overlap with one another. ORFs predicted by 3 or more tools to be GPCRs were then gathered for phylogenetic and structural analysis.

For the phylogenetic analysis, we used T-Coffee [39] for the multiple sequence alignment, the Clustal Omega-phylogeny web tool [40] http://www.ebi.ac.uk/Tools/msa/clustalo/ for the tree generation using a Neighbor-joining approach with default parameters, and FigTree (http://tree.bio.ed.ac.uk/software/figtree) for visualizing the tree data. Furthermore, we selected a subset of ORFs for tertiary structure homology modeling using RaptorX [41] found at http://raptorx.uchicago.edu/StructurePrediction/predict/. For each submitted ORF, RaptorX identified its best structure template, reported the percentage of residues modeled, and computed a p-value for the fit. We manually screened each structure model, counted the number of predicted helices *h* and gave it a signed score +*h* or -*h*. A positive score would indicate that all *h* helices are intact (i.e., not broken in the middle) and the overall structure visually resembled the well-established 3D GPCR structures published in GPCRDB (http://gpcrdb.org/). Structures not satisfying these criteria received a score of–*h* instead. With this scheme, an ORF with score +7 would be a strong candidate for a full-length GPCR, and a score between +3 and +6 may indicate a partial GPCR. Based on these scores, we selected our final list of predicted putative GPCRs.

## Results and discussion

### The *R*. *australis* foreleg transcriptome

Approximately 7 μg of DNA-free RNA was produced from the foreleg tissues and a total of 283,819 raw sequencing reads were assembled into 4,888 unigenes. Following trimming and artifact elimination, 4,782 unigenes was compiled (S1 File) and submitted to GenBank (Accession Number GEMR00000000).

**Fig 2:**
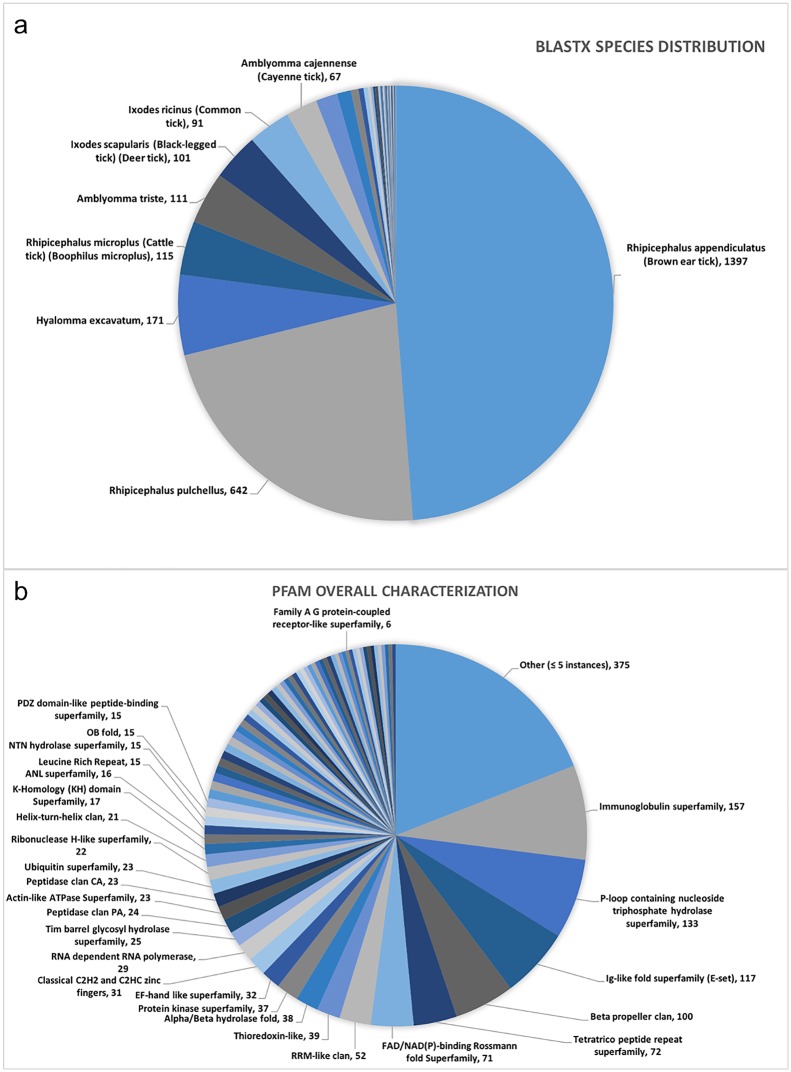
*R*. *australis* foreleg transcriptome annotation. a) Top Blastx hits by species; b) Characterization of Pfam predictions for all ORFs with at least 50 amino acids. The chart represents those ORFs that had a significant hit in Pfam and belonged to a clan/superfamily. Clans/superfamilies containing less than 5 sequences were grouped in the “others”.

The unigenes range in length from 200 to 10,397 nucleotides, with a mean and standard deviation of 920 ± 669. When these unigenes were characterized using Blastx and the Uniref100 database, a total of 2,923 (61%) had a significant hit (e-value 1e-06) (S3 File). Approximately 98% of the hits were to a tick sequence, of which 76% corresponded to *Rhiphicephalus*, 10% to *Amblyomma* and 7% to *Ixodes*. The most prevalent species was *Rhipicephalus appendiculatus* (48.74%) followed by *Rhipicephalus pulchellus* (22.40%). The species distribution is displayed in Fig 2a. Later, we describe transcripts annotated as encoding GPCR-related proteins. These include protein kinase A, G proteins, guanine nucleotide exchange factor, GTPase-activating proteins, adenylcyclase, and arrestin. This foreleg transcriptome also contains transcripts annotated by Blastx as encoding aquaporins (6), various transporters (69), cytochrome P450s (15), various receptors (52), cuticle constituents (4), among others. There were also 536 annotated as encoding uncharacterized proteins. Thus, of the 4,782 transcripts, 2,295 (48%) either had no significant sequence similarity or had a hit to an uncharacterized protein.

All unigenes were tested by the ORF finder script, and 40,907 ORFs with ≥50 amino acids were identified (S4 File). Using Pfam we were able to retrieve a list of 3,062 ORFs that were significantly aligned to at least one family in the Pfam database (http://pfam.xfam.org/help#tabview=tab5) with E-value ≤ 1 or bit score > 0. Among these, 1,967 were found belonging to a clan and a total of 78 different clans were involved. The pie chart in Fig 2b displays the clan or superfamily characterization. The “Immunoglobulin superfamily” was the most abundant (157 ORFs), followed by “P-loop containing nucleoside triphosphate hydrolase superfamily” (133 ORFs), and “Ig-like fold superfamily (E-set)” (117 ORFs). Each of these clans contains over 5% of the significant hits with identified clans. Other clans of interest included ABC transporters (7 ORFs), cystatin-like (8 ORFs), drug metabolite transporter (10 ORFs), ion channel (6 ORFs) and major facilitator (12 ORFs).

Comparing the foreleg transcriptome clan distribution to that of the *R*. *microplus* synganglion [6], we found that all except one of the foreleg transcriptome clans were also found in the synganglion. This exception was the NifU C-terminal domain-like superfamily, which contained two ORFs expressed in the forelegs, but none from the synganglion. On the other hand, there were 113 clans in the synganglion transcriptome, containing 539 ORFs, that were absent in the foreleg transcriptome. These clans are listed on the “PfamForelegVsSyngComparison” tab in S3 File. Among them, the Galactose-binding domain-like superfamily contained 39 ORFs with this domain but none were found in the foreleg ORF dataset.

We also noted that 8 out of the top 10 clans for these two cattle tick transcriptomes were in common. They were the Beta-Propeller clan, P-loop containing nucleoside triphosphate hydrolase superfamily, Immunoglobulin superfamily, Ig-like fold superfamily (E-set), Tetratrico peptide repeat superfamily, FAD/NAD(P)-binding Rossmann fold Superfamily, RRM-like clan, and Protein kinase superfamily. However, the Classical C2H2 and C2HC zinc fingers and Actin-Like ATPase Superfamily, ranked 4^th^ and 10^th^ respectively in the synganglion transcriptome, were ranked 12th and 16th in the foreleg transcriptome. At the same time, the clans Thioredoxin-like and Alpha/Beta hydrolase fold, which were 8th and 9th in the foreleg dataset, were 18th and 21st in the synganglion.

### GPCR prediction

Table 2 lists all the unigene ORFs predicted to encode GPCRs by the sequence alignment methods, and S5 File contains details of the predictions. Only 4 ORFs were predicted to encode GPCRs by the Blastx annotation of the foreleg transcriptome. Blastp searches of our predicted ORF dataset against the predicted GPCRs of *I*. *scapularis* [32], the predicted neuropeptide and neurohormone GPCRs [33–34] of *T*. *urticae* [33] and a diverse collection of other chelicarates [34], and the predicted GPCRs of the *R*. *microplus* and *R*. *australis* synganglion transcriptomes [6] yielded another 14 significant alignments with E-value < 1e-06, respectively. In the Pfam analysis, only 6 sequences aligned to the 7tm families. All 6 sequences belonged to the “Family A G protein-coupled receptor-like superfamily.” Three of the sequences hit to the secretin family, 1 to the rhodopsin-like family A, and 2 to the lung 7tm receptor family.

**Table 2:** ORFs predicted as GPCRs by any of the alignment-based tools.

Foreleg ORF	Blastx vs Uniref100	Blastp vs *T*.*urti*	Blastp vs Chelicarates	Blastp vs Syng	Blastp vs *I*.*scap*	Pfam
athaller3876_117(length)_1(strand)_1(frame)				**x**		
athaller1224_421(length)_1(strand)_0(frame)				**x**		
athaller3802_175(length)_1(strand)_2(frame)				**x**		
athaller3787_156(length)_1(strand)_0(frame)				**x**		
athaller1175_662(length)_1(strand)_1(frame)			**x**		**x**	**x**
athaller1230_465(length)_-1(strand)_0(frame)					**x**	
athaller2715_233(length)_1(strand)_2(frame)		**x**	**x**		**x**	
athaller2824_126(length)_1(strand)_1(frame)					**x**	
athaller356_291(length)_1(strand)_1(frame)	**x**				**x**	**x**
athaller357_301(length)_1(strand)_2(frame)	**x**					**x**
athaller4258_129(length)_-1(strand)_2(frame)		**x**	**x**		**x**	
athaller4697_102(length)_1(strand)_0(frame)			**x**		**x**	**x**
athaller675_193(length)_1(strand)_0(frame)					**x**	
athaller2474_190(length)_1(strand)_1(frame)	**x**					**x**
athaller4147_161(length)_-1(strand)_2(frame)	**x**					**x**
athaller1305_187(length)_-1(strand)_1(frame)		**x**	**x**			
athaller1897_360(length)_-1(strand)_2(frame)		**x**	**x**			
athaller508_240(length)_-1(strand)_1(frame)		**x**	**x**			

The SVM-based GPCRpred predicted 111 GPCR candidates (S6 File). Based on the IUPHAR GPCR nomenclature system, 101 of them belonged to class A, 3 to class B, 4 to class C, and 3 to class D. For the 101 predicted class A GPCR’s, GPCRpred also indicated their subfamilies: 12 were Rhodopsins, 3 were in the Olfactory subfamily, and the remaining 86 were assigned to subfamilies that pertain to the type of ligand that they bind to: 58 peptides, 18 amines, 1 hormone-protein, 2 prostanoid, 4 nucleotide-like and 3 lysospingolipid.

A total of 172 ORFs were predicted by the TMHMM approach to be either a full-length or partial candidate GPCR. Using the criteria described in the methods, we classified 14 ORFs as full-length, 33 as possible full-length and 125 as partial candidate GPCRs (see S7 File).

There were 251 ORFs predicted by at least one of the methods above (S8 File). These ORFs were further screened by PCA-GPCR, resulting in 176 GPCR predictions (S9 File), with 152 classified as Class A (Subfamilies: 14 (Rhod)opsin, 13 Amine, 16 Class A Orphan/other, 2 Hormone protein, 4 Lysosphingolipid & LPA (EDG), 3 Melatonin, 21 Nucleotide-like, 23 Olfactory, 54 Peptide, 1 Prostanoid and 1 Thyrotropin-releasing hormone & Secretagogue), 10 class B (Subfamilies: 5 GPR133, 1 GPR64, 1 ERM1, 1 Brain-specific angiogenesis inhibitor, 1 Calcitonin and 1 Latrophilin), 8 class C (Subfamilies: 4 GABA-B, and 4 Class C Other) and 6 Vomeronasal (Subfamilies: 4 Vomeronasal receptors V1RL, 1 Vomeronasal receptors V1RJ & VIRK and 1 Vomeronasal receptors V1RJ).

**Fig 3:**
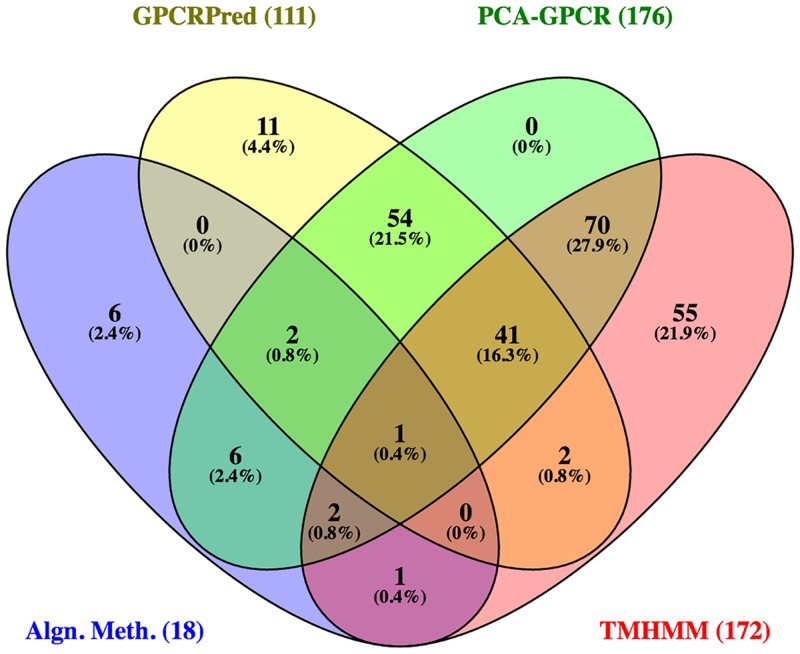
Venn Diagram showing numbers of GPCRs predicted by 4 different approaches: Alignment methods (Blast and Pfam), TMHMM, GPCRpred, and PCA-GPCR. The number of total predictions by each approach is indicated in parenthesis.

Fig 3 shows the numbers of GPCR predictions by the different methods and their overlap. The alignment methods produced the least number of positive predictions, probably because there are relatively few known GPCR sequences for ticks to date, and some families of GPCRs do not always exhibit high sequence similarities. TMHMM predicted the highest number of GPCRs. This was expected because our TMHMM approach allowed partial GPCRs to be included in the prediction.

**Fig 4:**
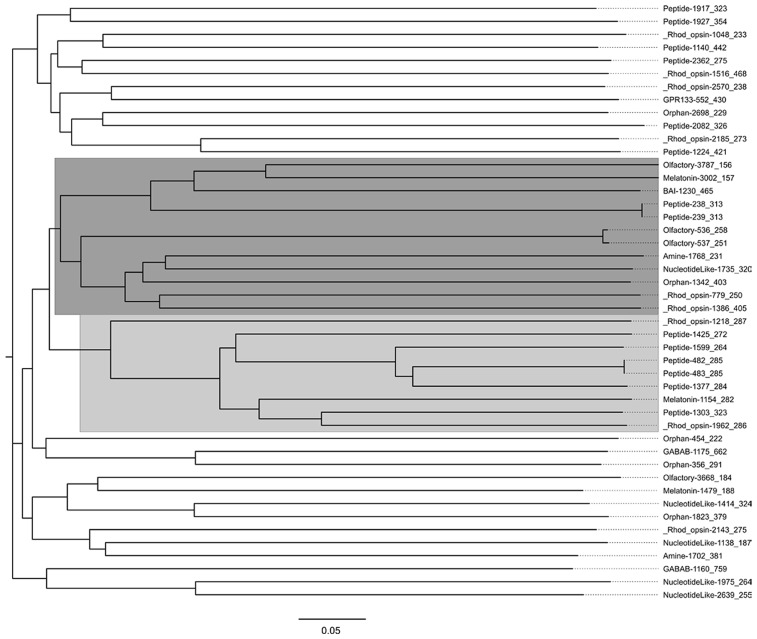
Phylogenetic tree of the ORFs that were predicted by 3 or more approaches. Clades containing clusters of ORFs from the same GPCR subfamilies are highlighted.

While wet lab confirmation is necessary to verify *in silico* predictions and to directly study activity and ligand specificity, higher confidence in GPCR prediction can be placed on ORFs predicted by multiple methods to encode GPCRs. This would be especially true when predictions are positive by both sequence alignment- and structural similarity-based protocols. Table 3 lists 46 ORFs from the *R*. *australis* foreleg transcriptome that were predicted to encode GPCR’s by at least 3 different approaches. The phylogenetic tree in Fig 4 displays the extent of similarities between these ORF sequences. (For compactness of the display and easier recognition, we shorten the names of the ORFs at this point to show its subfamily classification by PCA-GPCR, followed by their unigene numbers and lengths.) Despite the lack of sequence homology among classes, each GPCR class has its own characteristic set of conserved amino acids motifs that allow for multiple sequence alignment and consensus sequence generation [42]. It was interesting to observe that 3 of the 4 olfactory GPCRs predicted by PCA-GPCR were grouped together in the dark-grey cluster and that 6 of 9 predicted GPCRs in the light-grey cluster belong to the peptide subfamily.

**Table 3:** ORFs predicted to be GPCRs by at least 3 approaches.

ORF	Pred. by^1^	PCA-GPCR Class	PCA-GPCR Subfamily	GPCRpred Class	GPCRPred Subfamily
**athaller1048_233(length)_-1(strand)_0(frame)**	**TGP**	**A**	**(Rhod)opsin**	**A**	**Rhodopsin**
**athaller1218_287(length)_1(strand)_2(frame)**	**TGP**	**A**	**(Rhod)opsin**	**A**	**Rhodopsin**
athaller1386_405(length)_1(strand)_0(frame)	TGP	A	(Rhod)opsin	A	Prostanoid
athaller1516_468(length)_-1(strand)_0(frame)	TGP	A	(Rhod)opsin	A	Amine
athaller1962_286(length)_1(strand)_0(frame)*	**TGP**	**A**	**(Rhod)opsin**	**A**	**Rhodopsin**
athaller2143_275(length)_1(strand)_0(frame)	TGP	A	(Rhod)opsin	A	Peptide
**athaller2185_273(length)_-1(strand)_2(frame)**	**TGP**	**A**	**(Rhod)opsin**	**A**	**Rhodopsin**
athaller2570_238(length)_-1(strand)_0(frame)	TGP	A	(Rhod)opsin	A	Peptide
**athaller779_250(length)_1(strand)_1(frame)**	**TGP**	**A**	**(Rhod)opsin**	**A**	**Rhodopsin**
athaller1702_381(length)_-1(strand)_2(frame)	TGP	A	Amine	A	Peptide
athaller1768_231(length)_-1(strand)_2(frame)	TGP	A	Amine	A	Peptide
athaller1154_282(length)_1(strand)_0(frame)	TGP	A	Melatonin	A	Peptide
athaller1479_188(length)_1(strand)_2(frame)	TGP	A	Melatonin	A	Peptide
athaller3002_157(length)_-1(strand)_1(frame)	TGP	A	Melatonin	A	Peptide
athaller1138_187(length)_1(strand)_2(frame)	TGP	A	NucleotideLike	A	Amine
athaller1414_324(length)_-1(strand)_2(frame)	TGP	A	NucleotideLike	A	Amine
athaller1735_320(length)_-1(strand)_1(frame)	TGP	A	NucleotideLike	A	Peptide
athaller1975_264(length)_1(strand)_0(frame)	TGP	A	NucleotideLike	A	Peptide
athaller2639_255(length)_1(strand)_2(frame)	TGP	A	NucleotideLike	A	Peptide
athaller1224_421(length)_1(strand)_0(frame)	AGP	A	Peptide	A	Peptide
athaller3668_184(length)_-1(strand)_1(frame)ǂ	TGP	A	Olfactory	A	Peptide
athaller3787_156(length)_1(strand)_0(frame)ǂ	TAP	A	Olfactory	NA	-
athaller536_258(length)_-1(strand)_2(frame)*	**TGP**	**A**	**Olfactory**	**A**	**Olfactory**
athaller537_251(length)_-1(strand)_2(frame)*	**TGP**	**A**	**Olfactory**	**A**	**Olfactory**
athaller1342_403(length)_-1(strand)_0(frame)	TGP	A	Orphan	A	Rhodopsin
athaller1823_379(length)_1(strand)_2(frame)	TGP	A	Orphan	A	Rhodopsin
athaller2698_229(length)_-1(strand)_1(frame)	TGP	A	Orphan	A	Rhodopsin
athaller356_291(length)_1(strand)_1(frame)	TAP	A	Orphan	NA	-
athaller454_222(length)_1(strand)_0(frame)	TGP	A	Orphan	A	Peptide
**athaller1140_442(length)_-1(strand)_0(frame)**	**TGP**	**A**	**Peptide**	**A**	**Peptide**
athaller1303_323(length)_-1(strand)_0(frame)	TGP	A	Peptide	A	Rhodopsin
athaller1377_284(length)_1(strand)_0(frame)	TGP	A	Peptide	A	Rhodopsin
**athaller1425_272(length)_-1(strand)_2(frame)**	**TGP**	**A**	**Peptide**	**A**	**Peptide**
**athaller1599_264(length)_1(strand)_0(frame)**	**TGP**	**A**	**Peptide**	**A**	**Peptide**
athaller1917_323(length)_-1(strand)_0(frame)*	**TGP**	**A**	**Peptide**	**A**	**Peptide**
athaller1927_354(length)_-1(strand)_2(frame)	TGP	A	Peptide	A	Nucleotide-like
**athaller2082_326(length)_-1(strand)_0(frame)**	**TGP**	**A**	**Peptide**	**A**	**Peptide**
**athaller2362_275(length)_-1(strand)_0(frame)**	**TGP**	**A**	**Peptide**	**A**	**Peptide**
**athaller238_313(length)_-1(strand)_2(frame)**	**TGP**	**A**	**Peptide**	**A**	**Peptide**
**athaller239_313(length)_-1(strand)_2(frame)**	**TGP**	**A**	**Peptide**	**A**	**Peptide**
**athaller482_285(length)_1(strand)_2(frame)**	**TGP**	**A**	**Peptide**	**A**	**Peptide**
**athaller483_285(length)_1(strand)_2(frame)**	**TGP**	**A**	**Peptide**	**A**	**Peptide**
athaller1230_465(length)_-1(strand)_0(frame)	AGP	B	Brainspecific angiogenesis inhibitor (BAI))	B	-
athaller552_430(length)_1(strand)_0(frame)	TGP	B	GPR133	A	Amine
athaller1160_759(length)_1(strand)_1(frame)	TGP	C	GABAB	A	Amine
athaller1175_662(length)_1(strand)_1(frame)**	**TAGP**	**C**	**GABAB**	**C**	-

**Boldface**: Sequences whose subfamilies were consistently predicted by PCA-GPCR and GPCRpred

* Sequences that had +6 and +8 in the RaptorX 3D model score

** This sequence was consistently predicted as GPCR by all methods.

^ǂ^ Possible olfactory GPCR, predicted to be partial GPCR by TMHMM.

^1^Tools that predicted the ORF to be a GPCR (T = TMHMM, A = AlignmentMethods, G = GPCRpred, P = PCA-GPCR)

**Fig 5:**
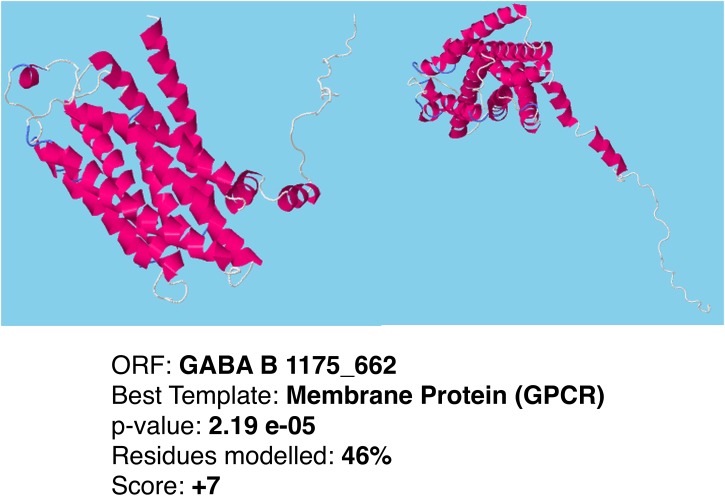
3D structure model for ORF athaller1175_662. Tertiary structure of a predicted GABA_B_ receptor. This ORF is consistently considered to be GPCR by all the prediction approaches used.

All 46 ORFs in Table 3 were predicted by both PCA-GPCR and GPCRPred, from which the GPCR classification and subfamily information are available in most cases. The subfamilies predicted by PCA-GPCR and GPCRpred are consistent with each other for 17 ORFs (boldfaced in Table 3), which include 10 Peptide-binding, 5 Rhodopsin, and 2 Olfactory type GPCRs. The ORF athaller1175_662 was the only one (also boldfaced) predicted by all 4 approaches, and it was considered Class C by both PCA-GPCR and GPCRpred. Noting the high level of consistency in the classifications of these 18 ORFs, we analyzed these 18 ORFs by the tertiary structure modeling tool RaptorX. The results are tabulated in S10 File. The 3D models of 5 ORFs, with scores between +6 and +8, further supported that they were likely full-length GPCRs. The information for these 5 ORFs is summarized here:

1. athaller1962_286. TMHMM analysis classified as "possible full length GPCR" with 7 helices. GPCRpred and PCA-GPCR predicted this ORF to be Class A rhodopsin-like. The RaptorX score was +8. However, the best matching structural template was “Membrane Protein (Oxidoreductase). Alignment methods did not find a hit to any known GPCR.
2. athaller536_258 and athaller537_251. These two ORFs had 97% sequence similarity. They were both predicted by GPCRpred and PCA-GPCR to be Class A Rhodopsin-like olfactory GPCRs. TMHMM predicted 6 helices and classified this ORF as "possible full length GPCR". However, alignment methods did not find a match to a GPCR. RaptorX matched this ORF it to the “Membrane Protein” structural template with score +7.
3. athaller1917_323. TMHMM analysis classified this ORF “possible full length GPCR” with 6 helices, while GPCRpred and PCA-GPCR predicted as Class A peptide binding. RaptorX score was +6 but the best matching structural template was “Membrane Protein (transporter).” No matches to GPCRs were found by alignment methods.
4. athaller1175_662. This ORF was predicted by all methods to be GPCR. The top Blastp hit was a putative GPCR in *I*. *scapularis* [32], and Pfam aligned the ORF to the 7tm_2 family. Both GPCRpred and PCA-GPCR predicted this ORF as Class C, and PCA-GPCR further classified it as a GABA B receptor. Our TMHMM analysis also classified the ORF as a "possible full length GPCR". The best matching structural template identified by RaptorX was “Membrane Protein (GPCR)” with score +7. Fig 5 shows the 3D model of this putative GPCR as predicted by RaptorX.

Furthermore, because the foreleg material contained the Haller’s organ structure and associated sensilla and is considered an olfactory organ, we are particularly interested in identifying as many olfactory receptors in *R*. *australis* as possible. We therefore also performed 3D modeling on 3 other ORFs in Table 3 that were classified as Olfactory only by PCA-GPCR but not GPCRpred: athaller357_231, athaller3668_184, and athaller3787_156. Among these ORFs, athaller3668_184 and athaller3787_156 both received a score of +5 (S10 File) and were predicted by our TMHMM analysis as partial GPCR. These are marked with “ǂ” in Table 3.

### GPCR-related proteins

When using Blastx and Pfam to identify GPCRs from our ORF collection, we took advantage of the resulting best hits to also identify other GPCR-related proteins by searching for protein names known to be associated with GPCRs (see Table 4). From the Blastx results we found 42 ORFs annotated as encoding GPCR-related proteins. These included 3 annotated as Protein Kinase A (PKA), 2 as G proteins, 1 as Guanine nucleotide exchange factors (GEF), 31 as GTPase-activating proteins (GAPs and GTPases), 4 as Adenylcyclases and 1 as arrestin. Pfam identified 5 ORFs as encoding GPCR-related proteins, including 3 GAPs, 1 GEF, and 1 arrestin. We present a combined list of GPCR-related proteins found from the Blastx and Pfam results in Table 5, anticipating this data may provide useful information for future investigations to help elucidate the downstream activities in GPCR signaling pathways.

**Table 4:** GPCR-related search terms. List of names and terms of proteins known to interact with GPCRs.

Search term	Protein type
**adenylate cyclase**	Adenylate Cyclase
**subunit**	G-protein subunits
**receptor kinase**	GRK
**cAMP-dependent protein kinase**	PKA
**GTPase**	GTPase
**GTPase activating protein**	GAP
**GAP**	GAP
**exchange factor**	GEF
**GEF**	GEF
**Rho**	GTPase and GAP
**Ras**	GTPase
**GTP-binding**	GTPase and GAP

**Table 5:** Predicted GPCR-related proteins. Cattle tick foreleg transcripts annotated as encoding proteins related to GPCR activity.

**BlastX hits**	
**PKAs**	
cAMP-dependent protein kinase regulator	athaller128
CAMP-dependent protein kinase catalytic subunit isoform 2	athaller1899
cAMP-dependent protein kinase regulator	athaller2981
**G-Proteins**	
Guanine nucleotide-binding protein subunit beta-2-like 1 protein	athaller853
Guanine nucleotide binding protein beta subunit	athaller2293
**GAPs & GEFs**	
Putative mitofusin 1 gtpase involved in mitochondrila bioproteinsis	athaller282
Putative mitofusin 1 gtpase involved in mitochondrila bioproteinsis	athaller284
Ras-like GTP-binding protein Rho1	athaller398
Ras-like GTP-binding protein Rho1	athaller399
Putative rac1 gtpase effector fhos	athaller500
Putative rac1 gtpase effector fhos	athaller501
Ypt/rab specific gtpase activating protein gyp6	athaller785
Ypt/rab specific gtpase activating protein gyp6	athaller786
Putative rhoa gtpase effector dia/diaphanous (Fragment)	athaller1192
Ras-related protein Rap-1A	athaller1263
Ras-related protein Ral-A	athaller1266
Putative rab subfamily protein of small gtpase (Fragment)	athaller1833
Rho GTPase-activating protein RICH2 (Fragment)	athaller2021*
Putative vesicle coat complex copii gtpase subunit sar1	athaller2065
GTP-binding nuclear protein	athaller2080
Rhoa gtpase effector dia/diaphanous (Fragment)	athaller2113
Putative ypt/rab-specific gtpase-activating protein gyp1	athaller2150*
Putative rac1 gtpase effector fhos	athaller2477
Putative ras-related protein rab-11a	athaller2532
Ras-related protein Rab-18	athaller2665
Ras-related protein Rap-2C	athaller2666
Putative gtpase rab14 small g protein superfamily	athaller2700
Ras-related protein Rab-1A	athaller2702
Rasgap sh3 binding protein rasputin	athaller2830
Putative rac1 gtpase effector fhos	athaller3014
Large subunit GTPase 1 (Fragment)	athaller3190
Rho gtpase binding protein	athaller3535
Putative gtpase rab2 small g protein superfamily	athaller3629
Ras-related protein Rab-24	athaller4154
Rhoa gtpase effector dia/diaphanous	athaller4155
Putative retinitis pigmentosa gtpase regulator b (Fragment)	athaller4427
**Adenyl Cyclases**	
Adenylate cyclase terminal differentiation specific	athaller184
Adenylate cyclase terminal differentiation specific	athaller185
Adenylate cyclase terminal differentiation specific	athaller186
Adenylate cyclase terminal differentiation specific	athaller187
**Arrestins**	
Beta-arrestin	athaller2087*
**Pfam hits**	
**GAPs & GEFs**	
RhoGAP	athaller2021_271(length)_-1(strand)_2(frame)*
RabGAP-TBC	athaller2150_313(length)_-1(strand)_2(frame)*
RhoGAP	athaller3118_219(length)_1(strand)_2(frame)
RhoGEF	athaller2000_339(length)_1(strand)_1(frame)
**Arrestins**	
Arrestin_C	athaller2087_258(length)_-1(strand)_1(frame)*

*Predicted by both BlastX and Pfam

### Bioinformatic approaches for predicting GPCRs

There have been many studies on GPCR prediction from genomic or transcriptomic sequences in different organisms. These works typically involved alignment methods either as stand alone procedures or in combination with other approaches as the basis for GPCR prediction. Often, there are limited sequence similarities between GPCRs and this can hamper efforts to comprehensively identify the full complement of GPCRs, especially in newly sequenced species distantly related to well-annotated model species. The Blast and Blast-like approaches are most accessible and easy to use. However, as this study shows, where only 4 transcripts among the 4,782 unigenes of the *R*. *australis* foreleg transcriptome were identified as GPCR-encoding by Blastx, more complex methods can be required to comprehensively predict GPCRs. Our initial findings using Blastp against predicted GPCRs from the genomes and transcriptomes of *I*. *scapularis* and *R*. *microplus* found 11 additional ORFs encoding putative GPCRs. When further Blastp searches against predicted neuropeptide and neurohormone GPCR datasets from the spider mite and other chelicerates [33, 34] were performed, we identified 3 more ORFs that previous Blast searches had not discovered. These ORFs are noted in yellow highlights in the S5 File “BlastpVsSpiderMite” and “BlastpVsChelicerates” tabs, with ORF IDs of athaller1305_187(length)_-1(strand)_1(frame), athaller1897_360(length)_-1(strand)_2(frame), and athaller508_240(length)_-1(strand)_1(frame). These 3 new predicted GPCRs were not found by our TMHMM, GPCRpred, or PCA-GPCR approaches either. This points out the value of using as many specialized datasets as possible. On the other hand, these new predicted GPCRs only bring the total of sequence alignment-based predicted GPCRs from 15 to 18 (Table 2). The need exists for utilizing as many prediction approaches as feasible to acquire a comprehensive dataset of potential GPCRs. For example, Hill [43] combined sequence alignment with secondary structure prediction approaches to identify 276 GPCRs from the genome of the mosquito *Anopheles gambiae*. More recently, hidden Markov model (HMM) approaches were included to improve the predictions of the odorant-binding protein and chemosensory protein gene families in arthropods [44]. Campos et al. [45] constructed an HMM and SVM-based pipeline to classify and sub-classify GPCRs of the worms *S*. *haematobium* and *S*. *mansoni*. Guerrero et al. [6] adapted the HMM-based TMHMM tool for identifying transmembrane helices to construct a prediction scheme, which incorporated the use of ORF lengths as well as stop codon counts, to predict both full-length and partial GPCRs. We refined their procedure in this study, and complemented it with the additional non-sequence similarity alignment methods GPCRpred and PCA-GPCR. We included a phylogenetic analysis for the assessment of evolutionary distance among sequences within our basic set of predicted GPCRs, as done by Vieira et al. [44] and Campos et al. [45]. Because of the importance of topology assessment in GPCR prediction [46–47], we also constructed 3D models for selected GPCR candidates enabling the visualization of the signature 7 transmembrane helical regions present in GPCRs. The use of multiple bioinformatic tools based upon independent identification strategies allowed us to look for consistencies in predictions to identify high confidence GPCR candidates that can be the focus of wet lab verification.

## Conclusion and future development

Our investigation has identified novel tick GPCR candidates that can be prioritized for functional genomic studies to help elucidate tick physiology. Our dataset enables future studies to confirm the function and activity of these putative GPCRs, including their roles in signal transduction, host preference, and mate selection. Our prediction scheme was enhanced by using methods other than solely sequence similarity approaches and GPCR candidates were prioritized based upon multiple prediction outcomes, starting from raw transcript sequences and progressing to building 3D models. We are in the process of implementing a pipeline that incorporates all the tools used in this study into a web-based modular program, a prototype of which is available at http://GPCR.utep.edu.

## Supporting information

S1 File Contig sequences. Unigene fasta DNA sequences assembled from the 454 transcriptome sequences.
(FASTA)

S2 File Custom scripts. Customized ORF-finding scripts to: 1) examine all 6 open reading frames (ORFs) of each transcriptome unigene and output resulting ORFs ≥ 50 amino acids.
(DOCX)

S3 File Transcriptome overall characterization. Overall transcriptome characterization using Blastx and Pfam.
(XLSX)

S4 File ORFs. ORF sequences obtained from the ORF-finding script in S2 File.
(FASTA)

S5 File Alignment approach predictions. Alignment based GPCR predictions, including Pfam hits to 7tm-related families, Blastx hits from the foreleg transcriptome against UniRef100, Blastp hits from the foreleg transcriptome against GPCRs predicted from the *I*. *scapularis* [32] assembled genome, the synganglion transcriptomes of *R*. *microplus* and *R*. *australis* [6], *T*. *urticae* [33], and the chelicerates *Stegodyphus mimosarum*, *Latrodectus hesperus*, *Parasteatoda tepidariorum*, *Acanthoscurria geniculata*, *Mesobuthus martensii*, and *Dermatophagoides farinae* [34].
(XLSX)

S6 File GPCRpred predictions. Positive predictions from the GPCRpred.
(XLSX)

S7 File TMHMM predictions. GPCR predictions by TMHMM, grouped by length and stop codon criteria. Sheet 1: Full Length predictions, Sheet 2: Possible Full Length predictions. Sheet 3: Partial predictions.
(XLSX)

S8 File Combined results. Compilation of all unigene ORFs predicted to encode a GPCR by at least one predictive tool.
(XLSX)

S9 File PCA_GPCR output. PCA-GPCR output.
(XLSX)

S10 File 3D models. RaptorX 3D models for selected ORFs.
(XLSX)
