# Supplementary material for: Bioinformatic prediction of G protein-coupled receptor encoding sequences from the transcriptome of the foreleg, including the Haller’s organ, of the cattle tick, Rhipicephalus australis
Source: PLoS One. 2017 Feb 23;12(2):e0172326. doi: 10.1371/journal.pone.0172326 (PMC5322884; doi:10.1371/journal.pone.0172326)
Supplement: S2 File — Customized ORF-finding scripts to: 1) examine all 6 open reading frames (ORFs) of each transcriptome unigene and output resulting ORFs ≥ 50 amino acids. (DOCX) [file pone.0172326.s002.docx]

**Orffinder_seq.py**

#Adapted and modified from code found at: http://biopython.org/DIST/docs/tutorial/Tutorial.html#sec360

#Written in Python 2.7, requires Biopython

#Orffinder_seq.py writes possible protein coding regions to file from 6 open reading frames.

#Takes a fasta file as input, and outputs a text file containing the protein coding regions

#with an identifer using the previous file's descriptor and the length of protein,

#strand and frame of the given ORF

from Bio import SeqIO

#Output file

outputFile ='test.txt'

#Input file must be in FASTA format, no spaces between FASTA sequences

inputFile = 'orffindertest.fasta'

f=open(outputFile, 'w')

for record in SeqIO.parse(inputFile,"fasta"):

table = 1

min_pro_len = 50 #minimum length for protein coding regions

#This for loop runs twice for each sequence in the file

#strand represents from beginning of sequence, or from the end of the sequence (+1, or -1)

#nuc represents forward sequence and reverse complement

#First time it runs for a particular sequence, strand is +1, nuc is the forward sequence

#Second time it runs for that sequence, strand is -1, nuc is the reverse complement of that sequence

for strand, nuc in [(+1, record.seq), (-1, record.seq.reverse_complement())]:

for frame in range(3):

#The '*' represents the stop codons within the sequence

#Splits the ORF based on stop codons

for pro in nuc[frame:].translate(table).split("*"):

if len(pro) >= min_pro_len:

#Writing the descriptor information to file

f.write("\n" + ">" + record.description[0:13])

print("\n" +">" + record.description[0:13])

f.write("_%i(length)_%i(strand)_%i(frame) " \

% (len(pro), strand, frame))

print("_%i(length)_%i(strand)_%i(frame) " \

% (len(pro), strand, frame))

#Writing the protein sequence to a file

print ("\n" + pro)

pro1=str(pro)

f.write("\n" + pro1)

f.close()

**inputIntoTMHMM4**

#Written in Python 3.3

#Version 4.0

#(8/9/2013), added module to import with functions of commonly repeated functional

#(8/14/2013) added some error handling

#(8/14/2013) Refactoring code

import urllib #combined urllib and urllib in Python 3

import urllib.parse

import urllib.request

import math

import webutility #user written module

import re

import time

import InputErrorEx #user written module

#import random #for testing only

#************************

#VARIABLES THAT NEED TO BE MODIFIED:

#inputName -- filename of input file, change to your filename

#outputName -- this is the name of your output file

inputName = 'seqtestMod.txt'

outputName = '31notGPCRs.txt'

#error file

err = 'notGPCRsErrOut.txt'

#************************

#VARIABLES THAT MAY NEED MODIFICATION:

#Number of sequences that TMHMM accepts at a time

#Decrease this value if an error keeps getting thrown

numOfAccSeqs = 1800

#Don't change this one unless the URL to the TMHMM site changes

postURL = 'http://www.cbs.dtu.dk/cgi-bin/webface2.fcgi'

#**************************

#SECTION 1: Reading in the text file

#

#

#**************************

parsedFasta = webutility.parseFastaFile(inputName)

fastaList = parsedFasta[0]

print('Length of fastaList = ', len(fastaList))

seqIDList = parsedFasta[1]

seqList = parsedFasta[2]

#*************************

#SECTION 2: Preparing input for TMHMM

#

#

#*************************

numOfSeqs = len(fastaList)

seqTotal = numOfSeqs

inputList = []

currSeqNum = 0

print('seqTotal = ' , seqTotal)

print('numOfAcceptedSeqs = ', numOfAccSeqs)

while(seqTotal > 0):

if seqTotal <= numOfAccSeqs:

allowedNum = seqTotal

else:

allowedNum = numOfAccSeqs

seqTotal = seqTotal - allowedNum

inputData = ''

for di in range(0, allowedNum):

inputData = inputData + fastaList[currSeqNum]

currSeqNum = currSeqNum + 1

inputList.append(inputData)

wrt = open(outputName, 'w')

e = open(err, 'w')

il = 0

while(il < len(inputList)):

print("Iteration number = ", il)

data = {'configfile': '/usr/opt/www/pub/CBS/services/TMHMM-2.0/TMHMM2CONV.cf','SEQ': inputList[il], 'outform':'-short'}

if(il > 0):

print('Sleeping for 60 seconds!')

time.sleep(60)

try:

#if(random.random() < .5):

# webutility.raiseHTTPError(data, postURL)

tmhmmOut = webutility.inputToTMHMM(data, postURL)

outList = webutility.simpleParse(tmhmmOut)

if len(outList) < 4:

errMsg = "Error!! Reduce accepted number of sequences and restart"

raise InputErrorEx.InputError(errMsg)

for ol in range(0, len(outList)):

if(outList[ol][0:5].strip() == 'WARN'):

print('It had an issue with a sequence, check the errOutput.txt file')

e.write(outList[ol].strip() + '\n')

else:

wrt.write(outList[ol].strip() + '\n')

il = il + 1

except urllib.error.HTTPError:

print('An HTTP error occurred!')

e.write("HTTP Error occurred at iteration = ")

e.write(str(il) + '\n')

except InputErrorEx.InputError:

print(errMsg)

print(outList[0])

e.write(errMsg)

if(numOfAccSeqs > 1500):

numOfAccSeqs = numOfAccSeqs - 500

print("Curr value of numOfAcceptedSeqs = ", numOfAccSeqs)

except urllib.error.URLError:

e.write("URL Error this time")

wrt.close()

e.close()

**InputError(Exception)**

#Written in Python 3.3

#Very basic exception class

#Used for inputToTMHMM4.py

class InputError(Exception):

def __init__(self, value):

self.value = value

def __str__(self):

return repr(self.value)

**webutility**

#Author: Alexandria Ogrey (anogrey@miners.utep.edu)

#Written in Python 3.3

#Version 1.2

#Used with inputToTMHMM4.py

import urllib

import urllib.parse

import urllib.request

import urllib.error

import time

#This function requires that there are no blank lines at the beginning of the file or separating the FASTA sequences

#Input: Takes a file containing only FASTA sequences

#This method returns a list containing three lists: fastaList at [0], seqIDList at [1], and seqList at [2]

#fastaList contains the FASTA sequence, with the identifer and the sequence (as it was originally in the file)

#seqIDList contains a list that only contains the FASTA identifiers

#seqList contains a list that only contains the sequences

def parseFastaFile(filename):

f = open(filename, 'r')

fileList = f.readlines()

f.close()

fastaList = []

seqIDList = []

seqList = []

finalList = []

if (fileList[0][0:5] == "\n"):

print("There may be an error here!!!! Double-check your output from this function")

print("Remove the blank line from the beginning of the file")

for i in range(1, len(fileList), 2):

fasta = fileList[i-1] + fileList[i]

fastaList.append(fasta)

seqIDList.append(fileList[i-1])

seqList.append(fileList[i])

finalList.append(fastaList) #fasta identifer + sequence

finalList.append(seqIDList)

finalList.append(seqList)

return finalList

#getWebData is a method that handles the preliminary steps of passing data to a website

#See Python 3 documentation for more information

#data is the information that is passed to the website, postSite is the url for the website

def getWebData(data, postSite):

url_encoded = urllib.parse.urlencode(data)

byte_encoded = url_encoded.encode('utf-8')

req = urllib.request.Request(postSite, byte_encoded)

return req

#This method passes sequences to TMHMM and copies the results from the website it redirects to

def inputToTMHMM(data, postURL):

urllib.request.urlcleanup()

req1 = getWebData(data, postURL)

print('req1 = ', req1)

#TMHMM redirects, so these next few lines handle that

getRedirect = urllib.request.urlopen(req1).geturl()

print("getRedirect =", getRedirect)

time.sleep(60)

reqFinal = urllib.request.urlopen(getRedirect)

print('reqFinal = ', reqFinal)

outputFromWeb = reqFinal.read()

strOutput = outputFromWeb.decode('utf-8')

return strOutput

#This method was created to generate an HTTP error for testing purposes

def raiseHTTPError(data, url):

req = getWebData(data, url)

getSite = urllib.request.urlopen(req)

raise urllib.error.HTTPError(url, "dontknow", "error", "", getSite)

#This method splits the number of FASTA data (sequences+identifers) into multiples of numOfAcceptedSeqs

#This is used so that large numbers of sequences can be passed to TMHMM, without having to worry about breaking them up

def fastaSplitByNum(numOfAcceptedSeqs, fastaList):

numOfAccSeqs = numOfAcceptedSeqs

numOfSeqs = len(fastaList)

seqTotal = numOfSeqs #gets reduced

inputList = []

currSeqNum = 0

while(seqTotal > 0):

if seqTotal <= numOfAccSeqs:

allowedNum = seqTotal

else:

allowedNum = numOfAccSeqs

seqTotal = seqTotal - allowedNum

inputData = ''

for di in range(0, allowedNum):

inputData = inputData + fastaList[currSeqNum]

currSeqNum = currSeqNum + 1

inputList.append(inputData)

print(len(inputList))

return inputList

#Breaks up a file into lines

#File needs to already be opened

#Input: inputData is the variable containing the open file data

#Output: Returns each line of the file in a list called outputList

def simpleParse(inputData):

import re

reg = re.compile('\n[a-zA-Z].+')

outputList = reg.findall(inputData)

return outputList

#Parses each line of the file based on a regular expression

#Input: name of the file to be parsed (filename), and a regular expression (regex)

#Output: a list containing parsed lines of the file

def simpleParseRegex(filename, regex):

import re

f = open(filename, 'r')

fileContents = f.read()

reg = re.compile(regex)

outputList = reg.findall(fileContents)

f.close()

return outputList

#This method is the same as simpleParse, but allows for passing of a regular expression

#File needs to be already opened

#Input: inputData is the variable containing the open file data

#Output: Returns a list of the instances that match the regular expression

def parseRegex(filedata, regex):

import re

reg = re.compile(regex)

outputList = reg.findall(filedata)

return outputList

#Utilized for testing purposes

def checkFirstListItem(listName):

print(len(listName))

if(len(listName) > 1):

print(listName[0])

**Stop_Codon.py**

#Author: Anastasia Kellogg ([akellogg@alumni.colostate.edu](mailto:akellogg@alumni.colostate.edu))

“””

This is the stop codon analysis script.

It performs the length test first to determine if a sequence is full/possibly-full length

Full/Possible length sequences are located in their translated contig and the up and downstream codons counted

Anything with at least 2 stop codons up and down-stream are parsed as full length.

All others are parsed as possible full length.

"""

from Bio import SeqIO

import re

#This input file is a FASTA file containing the contigs translated in all 6 frames

#The naming convention must be the same as the one in the orffile.

#EXAMPLE: ContigName_###(length)_###(strand)_###(frame)

gpcrAAfile = "translatedcontigs.fasta"

#All GPCR candidates as determined by the TMHMM analysis

#Must be in FASTA format

orffile = 'GPCRs.fasta'

#Parsed FASTA output files

output1 = 'fullLenGPCR.fasta'

output2 = 'possibleGPCR.fasta'

output3 = 'notFullLengthGPCR.fasta'

#Parsed CSV output files

output4 = 'fullLenExcel.csv'

output5 = 'possibleExcel.csv'

output6 = 'notFullLenExcel.csv'

fullfasta = open(output1, 'w')

possfasta = open(output2, 'w')

notfasta = open(output3, 'w')

fullexcel = open(output4, 'w')

possexcel = open(output5, 'w')

notexcel = open(output6, 'w')

#Initiates dictionary

seq={}

#Reads sequence ids as keys for dictionary

#Maps ORF sequences to the ID keys

with open(orffile,'r') as orf:

for record in SeqIO.parse(orf,"fasta"):

seq[record.id]=[record.seq]

#Reads in translated contigs and matches to dict keys

#Adds the translated contigs into the dictionary list

with open(gpcrAAfile,'r') as contigAA:

for record in SeqIO.parse(contigAA,"fasta"):

seq[record.id].append(record.seq)

##seq[key][0] is ORF pertaining to GPCR

##seq[key][1] is the translated contig containing the GPCR ORF

#For each GPCR candidate

for key in seq:

orf=str(seq[key][0])

contig=str(seq[key][1])

if len(orf)<235:

#Finds the ORF's location in the translated contig

m=re.search(orf,contig)

#Defines upstream sequence

upstream=contig[:m.start()]

#Defines downstream sequence

downstream=contig[m.end():]

#Counts stop codons in upstream sequence

up_stop=upstream.count('*')

#Counts stop codons in downstream sequence

down_stop=downstream.count('*')

#If a short sequence is complete this line of code discards it

if up_stop>=2 and down_stop>=2:

pass

else:

#writes FASTA file

notfasta.write(">"+key+"\n")

notfasta.write(orf+"\n")

#writes EXCEL file

line=key+","+"NA"+","+"NA"+"\n"

notexcel.write(line)

else:

#Finds the ORF's location in the translated contig

m=re.search(orf,contig)

#Defines upstream sequence

upstream=contig[:m.start()]

#Defines downstream sequence

downstream=contig[m.end():]

#Counts stop codons in upstream sequence

up_stop=upstream.count('*')

#Counts stop codons in downstream sequence

down_stop=downstream.count('*')

#Checks if sequence is full length by counting stop codons

if up_stop>=2 and down_stop>=2:

#Writes FASTA file

fullfasta.write(">"+key+"\n")

fullfasta.write(orf+"\n")

#Writes EXCEL file

line=key+","+str(up_stop)+","+str(down_stop)+"\n"

fullexcel.write(line)

else:

#Writes FASTA file

possfasta.write(">"+key+"\n")

possfasta.write(orf+"\n")

#Writes EXCEL file

line=key+","+str(up_stop)+","+str(down_stop)+"\n"

possexcel.write(line)

fullfasta.close()

possfasta.close()

notfasta.close()

fullexcel.close()

possexcel.close()

notexcel.close()
